# Anticancer effects of gossypetin from *Hibiscus sabdariffa* in oral squamous cell carcinoma

**DOI:** 10.1590/1678-7757-2023-0243

**Published:** 2023-10-09

**Authors:** Ke HUANG, Zhibin LIU, Myoung-Ok KIM, Ki-Rim KIM

**Affiliations:** 1 Kyungpook National University Graduate School of Science and Technology Department of Dental Hygiene Sangju Republic of Korea Kyungpook National University, Graduate School of Science and Technology, Department of Dental Hygiene, Sangju 37224, Republic of Korea.; 2 Kyungpook National University Research Center for Horse Industry Department of Animal Science and Biotechnology, Sangju Republic of Korea Kyungpook National University, Research Center for Horse Industry, Department of Animal Science and Biotechnology, Sangju 37224, Republic of Korea.

**Keywords:** Apoptosis, Gossypetin, Oral squamous cell carcinoma, Proliferation

## Abstract

**Objective:**

Gossypetin, isolated from *Hibiscus sabdariffa* L, has been shown to have various pharmacological effects including anti-inflammatory and antibacterial activity against various diseases. However, since the effect of gossypetin in oral cancer remains to be reported, we aimed to investigate the anticancer activity and mechanisms of gossypetin in oral squamous cell carcinoma (OSCC).

**Methodology:**

The proliferation of OSCC cells was evaluated by cell viability and soft agar colony assays. The effects of gossypetin on the migration and invasion of OSCC cells was investigated by wound healing and transwell invasion assays, respectively. Apoptosis and cell cycle arrest were measured by flow cytometry. Moreover, the anticancer mechanism of gossypetin in OSCC cells was analyzed by western blotting.

**Results:**

Gossypetin inhibited the proliferation, migration, and invasion of OSCC cells and induced apoptosis by upregulating the Bax/Bcl-2 ratio and cell cycle arrest at the G2/M phase. Furthermore, gossypetin regulated the activation of extracellular signal-regulated kinase and nuclear factor-kappa B.

**Conclusion:**

Results showed that gossypetin inhibits the proliferation, migration, and invasion of OSCC cells and triggers apoptosis and cell cycle arrest in OSCC. Therefore, gossypetin has the potential for use as a chemopreventive agent in oral cancer.

## Introduction

Oral cancer is one of the most common cancers worldwide, 90% of which are oral squamous cell carcinoma (OSCC).^1,2^ Because OSCC is difficult to diagnose early and most patients are diagnosed with advanced cancer, they show a five-year survival rate below about 55%.^3,4^ Radiation therapy is successful in cases with early diagnoses but surgery is the first treatment course considered for advanced OSCC. A combination of surgery, radiation therapy, and chemotherapy is used for recurrent and metastatic OSCC.^5^ Although this combined treatment has improved the survival rate of patients with OSCC, their quality of life deteriorates, including changes in appearance due to surgery and a decline in oral physiological function due to radiation and chemotherapy. Difficulties swallowing and chewing (important functions of the oral cavity) can cause secondary nutritional problems that negatively affect patients’ entire body,^6-8^ thus requiring the development of preventive and therapeutic agents for oral cancer using safe natural products that have a low toxicity.

Genetic and phenotypic changes within cells deregulate the cell cycle and apoptosis mechanisms, resulting in cancer progression.^9^ Controlling these mechanisms can inhibit the occurrence and progression of cancer. Therefore, the main methods to control the development of oral cancer involve preventing abnormal cell proliferation, regulating the cell cycle, and inducing apoptosis.^10^

Phytochemicals have emerged as a promising approach to manage tumors.^11^ *Hibiscus sabdariffa* Linnaeus (Malvaceae), a plant native to Southeast Asia and tropical Africa, is commonly used as an ingredient in food or beverages due to its various pharmacological properties.^12^ Previous studies have reported that *H. sabdariffa* extracts induce gastric cancer cell apoptosis by the mitogen-activated protein kinase (MAPK) pathway.^13^ The active compound isolated from *H. sabdariffa* flowers mediated apoptosis by regulating Bcl-2 in human leukemia cells.^14^ *H. sabdariffa* leaf extracts also inhibited the invasion of human prostate cancer cells by regulating the Akt/nuclear factor-kappa B (NF-κB) signaling pathway.^15^

Gossypetin, a hexa-hydroxylated flavonoid in *H. sabdariffa*, shows various pharmacological activities, including antioxidant and antibacterial effects, and the amelioration of oxidative stress and DNA damage.^16-19^ Gossypetin has been found to have a beneficial effect by inhibiting the PDZ-binding kinase/T-LAK cell-originated protein kinase (PBK/TOPK) in skin cancer related to solar UV exposure.^20^ Moreover, gossypetin has shown a therapeutic effect on esophageal cancer by acting as a MAPK kinase3/6 inhibitor and suppressing prostate cancer by inducing apoptosis.^21,22^ However, the efficacy of gossypetin against oral cancer is yet to be studied. Thus, this study investigated the anticancer effects of gossypetin on the proliferation and apoptosis of OSCC cells.

## Methodology

### Regents and antibodies

**Figure 1:**
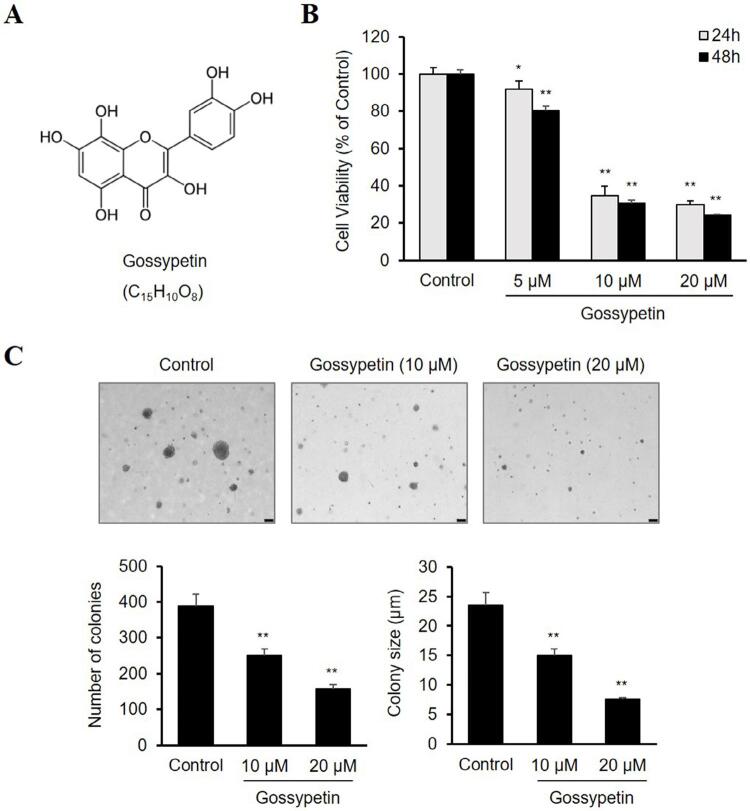
Effect of gossypetin on the proliferation of OSCC Ca9-22 cells. (A) Chemical structure of gossypetin. (B) Viability of Ca9.22 cells treated with various concentrations of gossypetin for 24 or 48 h as determined by the CCK-8 assay. (C) Representative photographs and quantitative analysis results of the colonization of Ca9-22 cells with or without gossypetin on soft agar. *p<0.01, **p<0.001 versus Control

Gossypetin (≥93% purity) was obtained from INDOFINE chemical Company (Hillsborough, NJ, USA) and dissolved in dimethyl sulfoxide (DMSO) (Figure 1A). Dulbecco’s modified Eagle medium (DMEM), phosphate-buffered saline (PBS), fetal bovine serum (FBS), antibiotic–antimycotic mixture containing 100 U/mL of penicillin and 100 μg/mL of streptomycin, and trypsin-ethylenediaminetetraacetic acid (EDTA) solution were purchased from Gibco BRL (Grand Island, NY, USA). Anti-Bax, anti-Bcl-2, anti-p21, anti-Cyclin B1, anti-Cyclin-dependent kinase 1 (CDK1), anti-p-p38, anti-p38, anti-p-NF-κB, and anti-NF-κB antibodies were obtained from Cell Signaling Technology (Danvers, MA, USA). Anti-actin, anti-p-Erk, and anti-Erk antibodies were purchased from Santa Cruz Biotechnology (Santa Cruz, CA, USA).

### Cell culture

The human OSCC cell line Ca9-22 was obtained from the Department of Oral Biology, Collage of Dentistry, Yonsei University (Seoul, Korea). Cells were cultured in DMEM with 10% FBS and 1% antibiotic–antimycotic mixture in a humidified atmosphere with 5% CO_2_ at 37°C.

### Cell viability assay

Ca9-22 cells (1×10^5^ cells/well) were seeded into 96-well plates and allowed to adhere overnight in the complete medium. The cells were then treated with various concentrations of gossypetin for 24 and 48 h. Viability of the Ca9-22 cells was assessed using the Cell Counting Kit-8 (CCK-8; Dojindo, Kumamoto, Japan). In brief, 10 μL of CCK-8 solution were added to each well and incubated for 2 h at 37°C. Absorbance was measured at 450 nm using a microplate reader (Thermo Fisher Scientific, Waltham, MA, USA).

### Soft agar colony formation assay

Ca9-22 cells (8×10^3^ cells/well) were suspended in complete growth medium with 0.6% agar and various concentrations of gossypetin in the base layer and 0.3% agar and gossypetin in the top layer. The plates were cultured at 37°C in 5% CO_2_ for two weeks. The colonies were analyzed and counted using Image-Pro Plus 6.0 (Media Cybernetics Inc., Rockville, MD, USA).

### Wound healing migration assay

The Ca9-22 cells were seeded into 24-well plates using the ibidi Culture-Insert 2 Well (ibidi, Gräfelfing, Germany) and allowed to grow to 90% confluence at 37°C in 5% CO_2_. The insert wells were then removed, and the cells were treated with various concentrations of gossypetin for 12 h. At 0 and 12 h, the scratched areas were photographed and measured using Image-Pro Plus.

### Transwell invasion assay

A transwell chamber (8-μm pore size, Corning Costar, Lowell, MA, USA) was used for the cell invasion assay. The Ca9-22 cells (2×10^4^ cells/well) were seeded into the upper chamber, which was precoated with Matrigel matrix (BD Biosciences, San Jose, CA, USA) in 100 μL DMEM with various concentrations of gossypetin. The lower chambers were filled with 600 μL DMEM containing 10% FBS. After incubation for 48 h, the cells were fixed in methanol for 20 min and the noninvaded cells on the upper surface of the membrane were removed with a cotton swab. The invaded cells on the lower surface of the membrane were stained with 0.5% crystal violet and counted using an inverted microscope.

### Flow cytometry analysis

Ca9-22 cells (1×10^6^ cells/well) were seeded into six-well plates, incubated overnight, and then treated with various concentrations of gossypetin for 48 h. The cells were harvested, washed with PBS, and fixed in 70% ethanol at −20°C for 24 h. For the apoptosis assay, cells were stained with annexin V-fluorescein isothiocyanate (FITC) and propidium iodide (PI) for 20 min in the dark at room temperature and analyzed using FACS Verse ﬂow cytometry (BD Biosciences). For the cell cycle assay, cells were incubated with FxCycle^TM^PI/RNase staining solution (Thermo Fisher Scientific) for 30 min and then analyzed by flow cytometry.

### Western blot analysis

Cells (1×10^6^) were plated into 100-mm dishes and treated with various concentrations of gossypetin for 24 h. Cells were lysed using PRO-PREP™ protein extraction solution (Intron Biotechnology, Seongnam, Korea), and the protein concentrations were measured using a BCA protein assay kit (Thermo Fisher Scientific). Equal amounts of protein samples were separated by sodium dodecyl-sulfate polyacrylamide (SDS-PAGE) gel electrophoresis and transferred to a polyvinylidene diﬂuoride (PVDF) membrane (Millipore, Billerica, MA, USA). The membranes were blocked with 5% skim milk in Tris-buffered saline containing 0.1% Tween 20 (TBST) at room temperature for 1 h and incubated with primary antibodies overnight at 4°C and then incubated with horseradish peroxidase (HRP)-conjugated secondary antibodies for 1 h at room temperature. Protein bands were detected with a SuperSignal™ West Pico Plus chemiluminescent substrate (Thermo Fisher Scientific) using an ImageQuant^TM^ LAS 500 imager (GE Healthcare Life Sciences, Marlborough, MA, USA).

### Statistical analysis

All results are shown as the mean ± standard error from at least three independent experiments. Statistical significance was determined using the Student’s *t*-test and one-way analysis of variance. A *p*-value<0.05 was considered statistically significant.

## Results

### Gossypetin inhibits the proliferation of Ca9-22 cells

To determine the effect of gossypetin on the proliferation of human Ca9-22 OSCC cells, cell viability and colony formation assay were performed. Figure 1B shows the results of the CCK-8 assay, in which the gossypetin treatment inhibited the viability of the Ca9-22 cells in a dose- and time-dependent manner. In particular, cell viability at 48 h totaled 80.5% at 5 µM, 30.8% at 10 µM, and 24.2% at 20 µM, showing a clear inhibitory effect under gossypetin concentrations totaling 10 µM or more (Figure 1B). A soft agar colony formation assay evaluated the effect of gossypetin on the anchorage-independent growth of Ca9-22 cells. Figure 1C shows that gossypetin reduced both the number and size of the colonies formed by the Ca9-22 cells in a dose-dependent manner.

### Gossypetin suppresses the migration and invasion of Ca9-22 Cells

**Figure 2:**
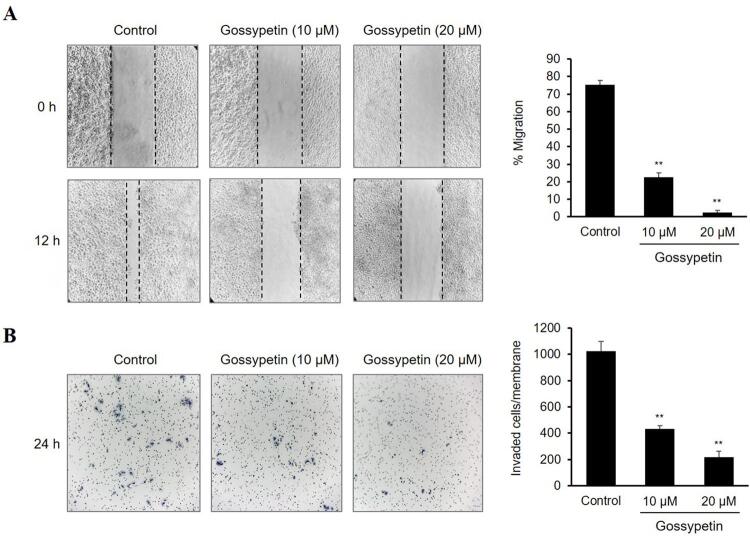
Effect of gossypetin on the migration and invasion of OSCC Ca9-22 cells. (A) Representative photographs and migration percentages of cells treated with or without gossypetin by a wound healing scratch assay. (B) Representative invasion photographs and numbers of cells treated with or without gossypetin by a Matrigel-coated transwell invasion assay. *p<0.01, **p<0.001 versus Control

Wound healing and transwell invasion assays assessed the inﬂuence of gossypetin on the migration and invasion of the Ca9-22 cells. Figure 2A shows that the monolayer migration of the control Ca9-22 cells without gossypetin treatment increased almost enough to close the wound after 12 h. The percentage of cell migration was 75.4±1.4% in the control group and 22.7±2.4 and 2.5±0.7% in 10- and 20-µM gossypetin groups, respectively (Figure 2A). The results of the transwell invasion assay showed that the number of Ca9-22 cells that invaded the Matrigel matrix and migrated through the membrane totaled about 1,022.7±77.0 in the control group and 434.0±23.1 and 216.3±47.1 in the 10- and 20-µM gossypetin groups, respectively (Figure 2B). Taken together, the gossypetin treatment remarkably suppressed both the migration and invasion abilities of the Ca9-22 cells in a concentration-dependent manner.

### Gossypetin induces G2/M cell cycle arrest in Ca9-22 cells

**Figure 3:**
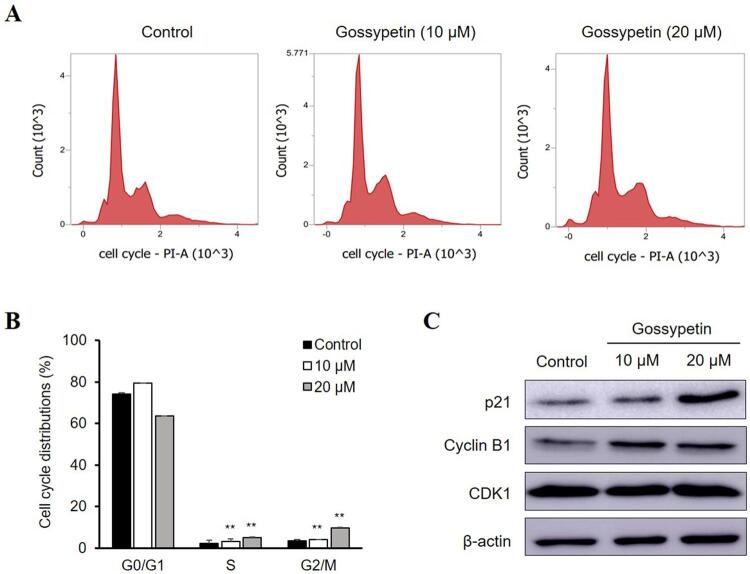
Effect of gossypetin on the G2/M cell cycle arrest of OSCC Ca9-22 cells. (A) Representative flow cytometry plots and (B) cell proportion at the G0/G1, S, and G2/M cell cycle phases in cells treated with or without gossypetin. (C) The expression of p21, cyclin B1, CDK1, and β-actin in cells treated with or without gossypetin by western blotting. *p<0.01, **p<0.001 versus Control

This study used flow cytometry to investigate the effect of gossypetin on the cell cycle and determine whether cell cycle changes were involved in the inhibition of the OSCC Ca9-22 cell growth by gossypetin. Treatment with 10 and 20 µM gossypetin increased the proportion of the Ca9-22 cells in the G2/M phase from 3.47±0.25% in the control untreated group to 4.16±0.53 and 9.67±0.29%, respectively (Figures 3A and B). As the assay confirmed that gossypetin induces the cell cycle arrest of Ca9-22 cells at the G2/M phase, the expression of the proteins related to the G2/M phase underwent subsequent assessment by western blot analysis. treatment with gossypetin increased the expression of p21 and cyclin B1 in a dose-dependent manner but CDK1 showed no significant changes (Figure 3C).

### Gossypetin induces apoptosis by increasing the Bax/Bcl-2 ratio in Ca9-22 cells

**Figure 4:**
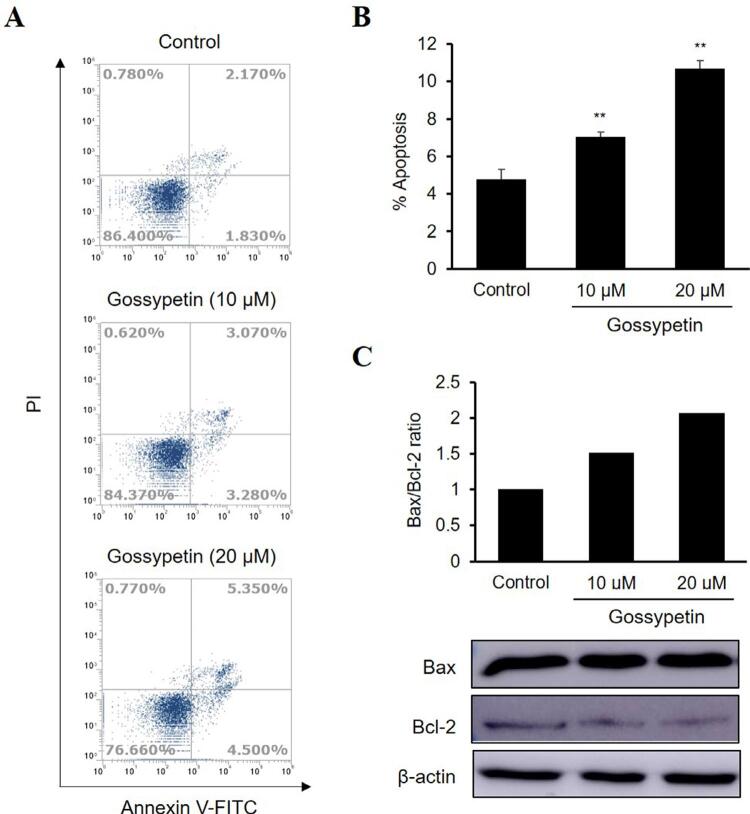
Effect of gossypetin on the apoptosis of OSCC Ca9-22 cells. (A) PI and annexin V-FITC staining and (B) percentages of apoptosis in cells treated with or without gossypetin by flow cytometry analysis. (C) Expression of Bax, Bcl-2, and β-actin in cells treated with or without gossypetin by western blotting and the ratio of Bax/Bcl-2 by densitometric analysis. *p<0.01, **p<0.001 versus Control

The effects of gossypetin on the apoptosis of OSCC Ca9-22 cells were confirmed by flow cytometry using annexin V-FITC and PI staining. Results showed that the apoptosis rate of the Ca9-22 cells treated with gossypetin increased in a dose-dependent manner compared with the control cells (Figure 4A). The apoptotic Ca9-22 cell population at 10- and 20-μM gossypetin concentrations increased to 7.05±0.97 and 10.69±1.52%, respectively, compared to the 4.79±1.14% in the control group (Figure 4B). To confirm the mechanism of the gossypetin-induced apoptotic effect, this study investigated the levels of the apoptosis-related proteins were investigated by western blot analysis. Treatment with gossypetin increased the expression of the Bax proapoptotic protein and decreased that of the Bcl-2 antiapoptotic protein, when compared to the levels of the control group (Figure 4C). In particular, the Bax/Bcl-2 ratio increased in a concentration-dependent manner by 1.5 and 2.1 times when treated with 10 and 20 μM of gossypetin, respectively, when compared to the control group.

### Gossypetin suppresses the phosphorylation of ERK1/2 and NF-κB in Ca9-22 cells

**Figure 5:**
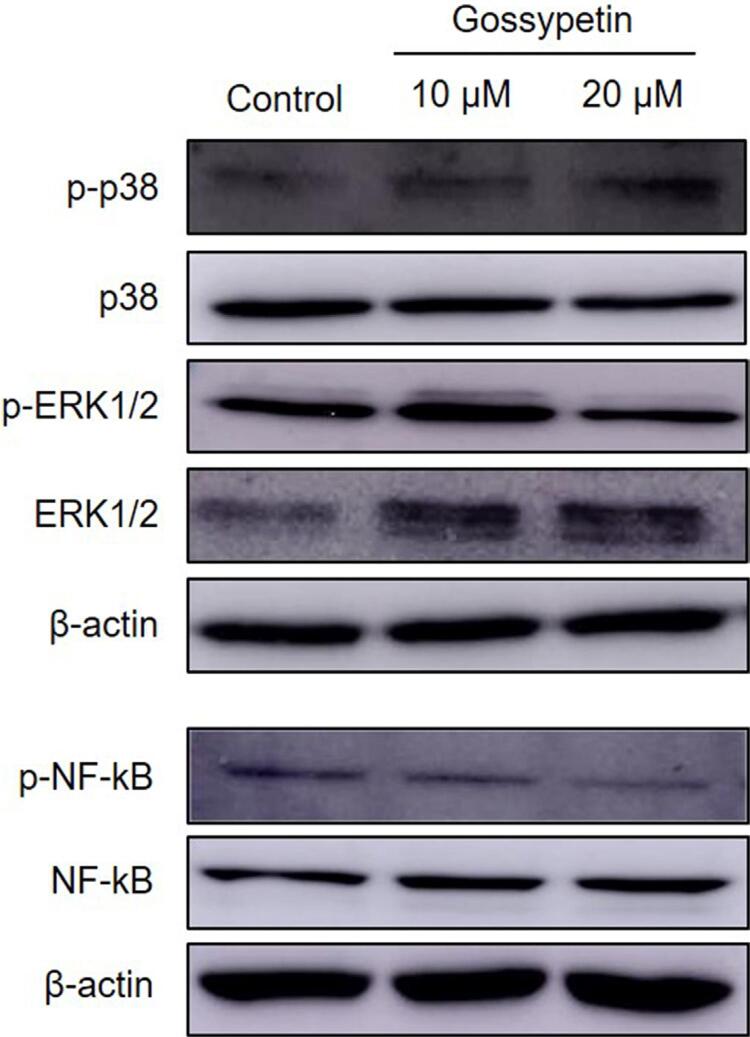
Effect of gossypetin on the phosphorylation of ERK1/2 and NF-κB of OSCC Ca9-22 cells. Expression of p-p38, p38, p-ERK1/2, ERK1/2, p-NF-κB, NF-κB, and β-actin in cells treated with or without gossypetin by western blotting

To identify the molecular mechanism of the anticancer effect of gossypetin in the OSCC Ca9-22 cells, we evaluated the activation of the MAPK and NF-κB signaling pathways play important roles in cancer progression. Figure 5 shows that the phosphorylation of ERK1/2 and NF-κB were markedly suppressed in the gossypetin-treated Ca9-22 cells compared with the control group. In particular, 20 μM of gossypetin effectively downregulated the expression of p-ERK1/2 and p-NF-κB. However, the expression of p38 MAPK showed no changes.

## Discussion

OSCC, which arises from the mucosal epithelial lining of oral cavities, accounts for >90% of all head and neck malignant neoplasms and has the highest morbidity and mortality.^2,3^ Although leukoplakia or erythroplakia are known to be clinically observed precancerous lesions, there are not many cases in which the early detection thereof prevents the progression to oral cancer.^3^ Moreover, since oral cancer often shows nonspecific early clinical symptoms, this type of cancer is highly likely to be misdiagnosed as another disease, making its detection challenging.^23,24^ When diagnosed with malignant oral cancer that has already metastasized to the surrounding tissues (such as the jaw or neck), extensive resection surgery and radiation therapy are performed, therefore the patients’ quality of life is drastically reduced.^25^ Thus, interest in cancer chemoprevention using natural products that have a low toxicity in relation to various systemic diseases as well as cancer is increasing, and health functional foods and medicines related to this are being actively developed. We investigated the inhibitory effect of gossypetin (a flavonoid isolated from the flower and calyx of *H. sabdariffa* known to have anti-inflammatory and antibacterial properties) on oral cancer.

Malignant tumors are characterized by rapid proliferation, migration, and invasive capacity, including extracellular matrix degradation.^26^ OSCC has an especially low survival rate due to its invasion into the jawbone and metastasis to the lymph nodes in the neck.^27^ OSCC occurs most commonly in the tongue, followed by the gingiva.^28^ Unlike those with a lingual epithelial origin, gingival epithelial-derived OSCC can invade the subgingival connective tissue and rapidly destroy the jawbone.^29^ As such, due to the complex tumor microenvironment of gingival epithelium-derived OSCC, we conducted a study on the efficacy of gossypetin in Ca9-22 cells, a squamous carcinoma of gingival origin.

Our results showed the antiproliferative activity of gossypetin against OSCC cells by confirming that gossypetin inhibited the survival rate and anchorage-independent growth of Ca9-22 cells in a concentration- and time-dependent manner. Moreover, we confirmed that gossypetin significantly inhibited the migration and invasion of Ca9-22 cells. These results indicate that gossypetin can prevent OSCC progression and metastasis by blocking cancer stages such as proliferation, migration, and invasion.

Cell cycle arrest and apoptosis, which represent DNA-damage responses to cancer cells, are useful anticancer strategies for various chemotherapeutic agents.^30^ We confirmed that gossypetin-induced G2/M cell cycle arrest in OSCC cells, which is supported by the finding that it induced the expression of the G2/M phase-related protein cyclin B1. In addition, it can be assumed that the upregulation of p21 expression by gossypetin caused this cell cycle blockade. However, there was no change in the expression of the tumor suppressor p53 in gossypetin-treated Ca9-22 cells. Interestingly, our results resemble a previous report that showed that the anthocyanin components isolated from *H. sabdariffa* markedly promoted G2/M arrest by inducing p21 in human leukemia cells.^31^ Collectively, gossypetin leads to the G2/M cell cycle arrest of OSCC cells by upregulating p21 expression in a p53-independent manner.

In OSCC Ca9-22 cells, treatment with gossypetin induced a tandem increase in annexin V and PI, indicating the induction of late apoptosis, as evinced by the regulation of Bax and Bcl-2 expression. Bcl-2 family proteins are composed of pro- and antiapoptotic proteins that can promote carcinogenesis and induce resistance to cancer treatment by activating apoptosis by regulating mitochondrial outer membrane permeability.^32^ Because mitochondria-mediated apoptosis is an important therapeutic strategy to improve oral cancer, the regulation of Bcl-2 family proteins is important.^33^ In particular, the ratio between Bax and Bcl-2 (which are major proteins of the Bcl-2 family) is considered an indicator of apoptosis susceptibility.^34^ We confirmed that the Bax/Bcl-2 ratio increased by more than twice that of the control group when treated with 20 µM of gossypetin. Therefore, these results suggest that gossypetin has potential as a chemopreventive agent for oral cancer as it is involved in the DNA damage of cancer cells by arresting cell cycles and inducing apoptosis.

In our results on MAPK and NF-κB signaling, 20 µM of gossypetin reduced the relative expression levels of p-ERK1/2/ERK1/2 and p-NF-κB/NF-κB by more than 50%, compared to that of the control group. The phosphorylation and activation of ERK1/2 mediates various cellular responses, such as cell proliferation, apoptosis, DNA repair, and cell cycles by regulating various molecules.^35^ The reduction of the p-ERK1/2 expression by gossypetin supports our findings, including proliferation inhibition, apoptosis induction, and cell cycle arrest in OSCC cells. NF-κB, a proinflammatory transcription factor that plays a pivotal role in the progression of most cancers, is also important in oral cancer.^36^ In a previous study, the activation of NF-κB induced the proliferation and inhibition of apoptosis in OSCC cells and promoted OSCC-induced mandible destruction in a mouse model.^37^ Our findings, including the proliferation inhibition and apoptosis induction of OSCC cells by gossypetin, can therefore be considered as anticancer effects related to the regulation of the ERK and NF-κB signaling pathways. Thus, the potential use of gossypetin as a chemopreventive agent for OSCC is suggested as a strategy to control these mechanisms. However, since this study is limited to an *in vitro* study conducted on only one OSCC cell line, additional efficacy evaluations on various cell lines and animal experiments is considered necessary for the practical use of gossypetin. Therefore, as a further study, we plan to conduct an *in vivo* study of the anticancer effect of gossypetin using an oral cancer-induced mouse model.

## Conclusion

We confirmed the anticancer effects of gossypetin against OSCC, a representative oral cancer. In the Ca9-22 OSCC cell line, gossypetin inhibited cell survival and anchorage-independent cell growth. Our results also showed that gossypetin significantly suppressed the migration and invasion of Ca9-22 cells according to the wound healing scratch and Matrigel-coated transwell invasion assays, respectively. Treatment with gossypetin-induced cell cycle arrest at the G2/M phase increased the expression of p21 and cyclin B1. Gossypetin also triggered apoptosis, which is supported by the increased changes in the ratio of proapoptotic Bax/antiapoptotic Bcl-2 proteins. These results of the gossypetin treatment of OSCC cells can be attributed to the regulation of ERK and NF-κB signaling activation. In conclusion, gossypetin, a flavonoid isolated from *H. sabdariffa*, has potential as a chemopreventive agent for oral cancer.
